# The development and validation of the Student Self-feedback Behavior Scale

**DOI:** 10.3389/fpsyg.2024.1495684

**Published:** 2025-01-06

**Authors:** Yongle Yang, Zi Yan, Jinyu Zhu, Wuyuan Guo, Junsheng Wu, Bingjun Huang

**Affiliations:** ^1^School of Education, Jingchu University of Technology, Jingmen, China; ^2^The Education University of Hong Kong, Hong Kong SAR, China; ^3^Haile Experimental School, Shenzhen, China

**Keywords:** self-feedback behavior, scale development and validation, Chinese student, cross validation, measurement invariance

## Abstract

Though the importance and benefits of students’ active role in the feedback process have been widely discussed in the literature, an instrument for measuring students’ self-feedback behavior is still lacking. This paper reports the development and validation of the Self-feedback Behavior Scale (SfBS), which comprises three dimensions (seeking, processing, and using feedback). The SfBS items were constructed in line with the self-feedback behavioral model. One thousand two hundred fifty-two high school students (Grade 10 to Grade 12) in mainland China participated in this survey. The exploratory factor analysis revealed a three-factor model reaffirmed in the confirmatory factor analysis. The multi-group CFA supported the measurement invariance of the SfBS across gender. Using the SfBS can help researchers and teachers better understand students’ self-feedback behavior and optimize benefits derived from the self-feedback process.

## Introduction

The shift of feedback research from a teacher-centered to a student-centered model has obtained more academic attention (Winstone et al., 2017; Bennett et al., 2017; Ryan and Deci, 2017; Mahoney et al., 2019). This shift highlights the role of student agency and how they could be more actively involved and eventually benefit from the feedback process to enhance their learning outcomes (Boud and Molloy, 2013; Dunworth and Sanchez, 2016). This study describes self-feedback behavior as students’ intentional behavior of seeking, processing, and using feedback for their learning improvement (Lipnevich et al., 2013; Lipnevich et al., 2016; Lipnevich and Panadero, 2021). Students could benefit from the self-feedback process since those who actively devote themselves to the self-feedback process would become more proficient in eliciting, making sense of information, and using the feedback for their learning improvement (Carless and Boud, 2018; Panadero et al., 2019a; Malecka et al., 2020; Yan and Carless, 2021). Furthermore, students who take more proactive agency in the self-feedback process likely become more proficient in their learning experiences and thus create more advanced learning opportunities for themselves. Eventually, they could gain more academic self-efficacy throughout the self-feedback process and achieve more outstanding academic achievement (Panadero et al., 2017; Van der Kleij, 2024).

Given the importance of self-feedback behavior, it is still understudied to measure students’ actions of seeking, processing, and using feedback (Evans, 2013; Winstone et al., 2017; Malecka et al., 2020). This instrument is expected to transform theoretical discussions into a shared understanding of students’ self-feedback behavior, thus advancing the research progressions of students’ engagement in the feedback process. This is the primary goal of the knowledge gap this study attempts to close.

## Literature review

### Behavior model of self-feedback

**Figure 1:**
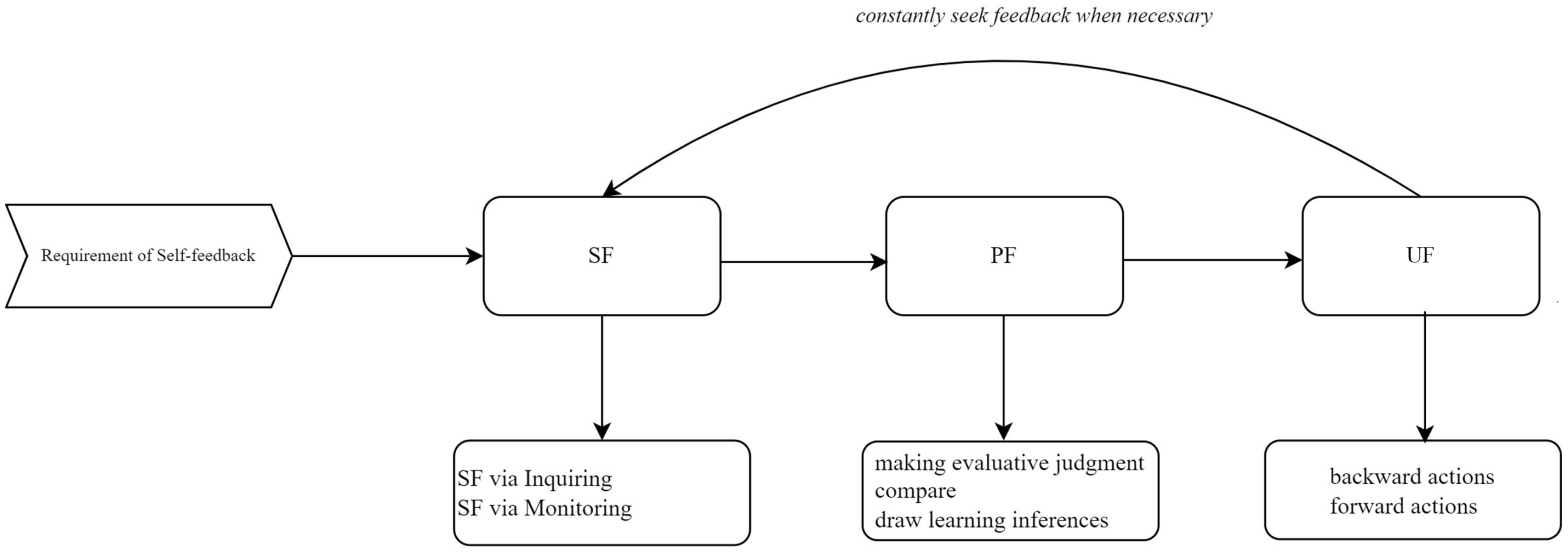
The self-feedback behavioral model. SF, seek feedback; PF, process feedback; UF, use feedback.

The shift of feedback studies to a “student-centered” framework has led many researchers to re-examine what students need to know to use feedback for their learning improvement. With this effort, Carless and Boud (2018, p. 1315) proposed feedback literacy, which “denotes the understandings, capacities, and dispositions needed to make sense of information and use it to enhance work or learning strategies.” However, students’ specific behaviors to engage in the feedback process have been surprisingly understudied (Molloy et al., 2019; Malecka et al., 2020), which limits its deserved potential to enhance students’ feedback learning. In this paper, unlike the conventional description of “self-level” feedback, which highlights students as the sole agents generating internal feedback without seeking input from external sources (Hattie and Timperley, 2007), we argue that self-feedback should highlight students’ active agency, and comprise three components: *students taking the active initiative to seek, process, and use the feedback information to improve their learning performance* (see Figure 1). Subsequently, each self-feedback action will be elaborated on.

### Seek feedback

Previous studies on feedback emphasize that students should actively seek feedback from external sources, positioning students as proactive participants in the feedback process (Winstone et al., 2017; Carless and Boud, 2018; Boud and Molloy, 2013; Yan and Carless, 2021). It is crucial for students to deliberately solicit comments from their physical learning environment and engage with relevant individuals (Nicol, 2021). There are two main behavioral methods for obtaining feedback: (a) inquiry, which involves directly seeking external input from teachers, peers, and friends, and (b) monitoring, which involves indirectly drawing explicit inferences from the environment, their past experiences, and their peers’ work (Carless, 2006; Leenknecht et al., 2019). This study borrows these two forms of feedback-seeking behavior, considering their appropriateness in the context of self-feedback.

### Process feedback

Once feedback is obtained, the following action shall be the feedback processing, as the received feedback information could often be mixed or conflicting from various sources and in different formats (Han and Xu, 2021). Students should, therefore, intentionally make evaluative judgments about the elicited comments (Nicol, 2014). However, making evaluative judgments about elicited comments can be emotionally and intellectually challenging, mainly when feedback originates from various sources, complicating its interpretation (McConlogue, 2015; Carless and Boud, 2018). To manage this complexity, students must develop the capacity to evaluate their work against the feedback received, using success criteria and exemplar work as benchmarks (Carless, 2015; Tai et al., 2017). This evaluative process is ideally enhanced through a meta-dialogue with teachers and peers, supplemented by additional learning resources (Carless and Boud, 2018; Panadero et al., 2019b). Subsequently, students can identify valuable inputs for their further use. The ultimate goal of feedback processing is to engage students in more profound reflection on their work by explicitly comparing it with previous coursework, learning objectives, and success criteria.

### Use feedback

Students need to take the initiative to use the feedback to maximize their learning attainments throughout the self-feedback process (Malecka et al., 2020; Molloy et al., 2019; Winstone et al., 2022; Wood, 2022; Dawson et al., 2023). Self-feedback action can often be considered short-term and long-term depending on the timing of follow-up actions taken. Short-term action is often correcting their mistaken work, summarizing their strengths and weaknesses, reflecting their learning attainment, and possibly calibrating the success criteria of their learning. These are all recommended practices to be embedded and operationalized in a curriculum setting (Malecka et al., 2020; Yan and Carless, 2021). Long-term action includes re-calibrating their learning goals, building self-feedback skills into their daily learning experiences, and eventually formulating individualized learning growth plans (Malecka et al., 2020; Molloy et al., 2019; Yan and Carless, 2021).

Lastly, the self-feedback process is not a one-off behavior. Instead, it should be a cyclical mode to enhance self-feedback effectiveness. Students should consistently practice self-feedback strategies during their learning process to maximize its potential benefits.

The relationship between self-feedback and these similar concepts was further explained, given its theoretical connections with internal feedback, self-assessment, and feedback literacy (Falchikov and Boud, 1989; Panadero et al., 2016; Li and Han, 2021). Nicol (2021) describes internal feedback as “the new knowledge that students generate when they compare their current knowledge and competence against some reference information” (p. 2), highlighting how students internalize external inputs through comparisons (Laudel and Narciss, 2023). However, self-feedback focuses on processing elicited external feedback to form students’ own learning inferences rather than on the internal generation of information. Self-assessment is defined as a process “during which students collect information about their performance, evaluate, and reflect on the quality of their learning process and outcomes according to selected criteria to identify their strengths and weaknesses” (Yan and Brown, 2017, p. 1248) emphasizes assessment accuracy, while self-feedback highlights actions taken upon processed feedback. Carless and Boud (2018) conceptualize feedback literacy with four key elements: appreciating feedback, making judgments, managing affect, and taking action (Molloy et al., 2019, p. 3), framing it as feedback capacity rather than behavioral engagement (Noble et al., 2020). Self-feedback, in contrast, emphasizes students’ behavioral engagement in the feedback process.

## Methods

### Instrument development

Adopting a theory-driven approach, the Self-feedback Behavior Scale (SfBS) was created in line with the self-feedback behavioral model described. Therefore, the SfBS is comprised of three sub-scales: seek feedback (SF), process feedback (PF), and use feedback (UF). The SfBS items were developed using two primary resources: adapted items from the Feedback Literacy Behavior Scale (Dawson et al., 2023) and the Self-assessment Practice Scale (Yan, 2018, 2020). Particularly, for the Seek Feedback (SF) and Use Feedback (UF) dimensions; the items were initially adapted from the corresponding dimensions from Feedback Literacy Behavior Scale given its theoretical connections, while the SF in SfBS emphasizing the seeking feedback behavior from various learning resources, and UF emphasizing the cyclical process for students to constantly seek external feedback for optimizing their learning improvement plan. For the Process Feedback (PF) dimension, items were generated initially through adaption from the dimension of Make Sense of Information (MS) from the Feedback Literacy Behavior Scale and Self-reflection (SER) from the Self-assessment Practice Scale, while the SfBS emphasizing the importance of making evaluative judgments about comments elicited from multiple sources, additionally, all original items generated based on three rounds of focus group discussions. The focus group members have different levels of feedback proficiency across various subject domains. An initial set of 26 items was created, but five were eliminated due to content redundancy. The 21 obtained items were then reviewed by six experts, three from the educational assessment field and three from veteran teachers. The expert panel evaluated the appropriateness of factor-item structure and the accuracy of item content. Four items were removed due to their ambiguity. After rounds of reviews, 17 items were eventually generated.

### The scale of seek feedback

Seeking feedback refers to students proactively soliciting comments from external sources. According to Ashford and Cummings (1983), this feedback can be named “seeking feedback through monitoring” and “seeking feedback through inquiry,” respectively. Henceforth, SF consists of six items to measure two types of feedback-seeking behavior: (a) seeking feedback through inquiry (4 items, e.g., *I ask people for feedback on certain elements of my work*); and (b) seeking feedback through monitoring (2 items, e.g., *When I am working on a task, I consider comments I have received on similar tasks*).

### The scale of process feedback

Process feedback refers to students evaluating the comments they received, considering the credibility of the sources of the comments, comparing the inputs they received with their prior experiences with similar works and future learning goals, etc. (Han and Xu, 2020). Therefore, PF comprises six items to measure students’ various feedback processing steps. Items (e.g., *When receiving conflicting information from different sources, I judge what I will use, and I carefully consider comments about my work before deciding if I will use them or not*) are curated to measure the actions students might take when they receive conflicting comments from different sources; they would like to make evaluative judgments about whether these comments are correct and relevant or not for their coursework before they would further consider it. Items (e.g., *When deciding what to do with comments, I consider the credibility of their sources*) are constructed to measure how students can distinguish the valuable information from non-useful or misleading information they received; Items (e.g., *I explicit the inferences after comparing comments with my leaning experiences*) are constructed to measure students’ practices of comparing the feedback with their past learning experiences of similar coursework.

### The scale of using feedback

Using feedback refers to how students use the processed information to facilitate their learning improvements. Therefore, the five-item scale (UF) measures how students used the comments and learning inferences they obtained to optimize their future learning strategy. Items (e.g., *I can formulate my learning improvement plan after explicit inferences*) aimed to measure whether students took measures to create their learning growth scheme after seeking and processing feedback information. Items (e.g., *I would spend more time working on my advantageous areas, and I would spend more time working on my weak areas*) were constructed to measure which areas the students took efforts to improve their learning attainment.

The scale was drafted in simplified Chinese to be administered to high school students in mainland China. It is a six-point positive Likert-type scale (1 = strongly disagree; 2 = disagree; 3 = slightly agree; 4 = agree; 5 = mostly agree; 6 = strongly agree) (Lam and Klockars, 1982). It aimed to match Chinese students’ inclination toward positive conformity, obtaining more variation in their responses (Brown and Harris, 2013).

### Data collection

The Human Research Ethics Committee (HREC) approval was acquired before the commencement of the investigation. Psychometricians and behavioral experts were consulted regarding the potential dangers and associated precautions. Before the survey started, a set of consent forms and information sheets was provided to relevant stakeholders. The participants from three high schools in mainland China were surveyed. A dataset of 1,252 students from Grade 10 to Grade 12 (aged 15–18 years) was collected in this study.

### Sample

The dataset was then randomly divided into two sub-samples: Sample 1 (*N* = 626) was used for a series of exploratory factor analysis (EFA) to obtain the factorial structure of self-feedback behavior. In contrast, Sample 2 (*N* = 626) was adopted for confirmatory factor analysis (CFA) to assess the model fit of the factorial structure. The demographics of participants in both samples were reported in Table 1.

**Table 1:** Demographics for the two samples.

	Sample 1		Sample 2	
	N	%	N	%
Male	348	55.59%	351	56.07%
Female	278	44.41%	275	43.93%
G10	212	33.87%	271	43.29%
G11	290	46.33%	237	37.86%
G12	124	19.81%	118	18.85%
Subtotal	626	100.00%	626	100.00%

### Data analysis

First, this study employed EFA with principal component extraction and oblique rotation (direct oblimin) in Sample 1 to delineate the factorial structure of the 17-item SfBS. Multiple criteria guided the factor extraction process. These consisted of scrutinizing the scree plot (Cattell, 1966; Floyd and Widaman, 1995), following Kaiser’s criterion, which entails extracting factors with eigenvalues equal to or exceeding 1.00 (Kaiser, 1960). Additionally, the commonalities of each variable, the proportion of variance explained, and the interpretability of the resultant factors were also evaluated. Furthermore, items demonstrating a disparity between their primary and secondary factor loadings of less than 0.20, alongside secondary factor loadings of at least 0.30, were identified as cross-loading items and subsequently excluded (Schaefer et al., 2015).

Second, CFA with maximum likelihood estimation was adopted in Sample 2 to compare the model fits between the theoretical and measurement structures resulting from EFA in Sample 1. As the model chi-square index is inclined to be significant and impacted by the sample size (Bentler and Bonett, 1980; Jöreskog and Sörbom, 1993; Newsom, 2012), other series of model fit indicators were considered, as reported in Table 2.

**Table 2:** CFA model fit indices for the two models on Sample 2.

	χ^2^	df	χ^2^/df	P	CFI	TLI	RMSEA	SRMR	AIC	BIC
Benchmark	/	/	< 3.00	<0.001	>0.90	>0.90	<0.08	<0.05	/	/
Model 1	127.536	41.000	3.111	<0.001	0.980	0.973	0.058	0.027	17139.668	17250.290
Model 2	160.030	51.000	3.138	<0.001	0.977	0.970	0.059	0.029	18557.948	18677.332

Third, this study assessed the validity of the SfBS using Messick (1995) framework, which treats validity as a comprehensive concept comprising six distinct aspects: content, substantive, structural, generalizability, external, and consequential validity (Messick, 1995). Moreover, the convergent and discriminant validities were examined to evaluate the item-factor structure of SfBS. Cronbach’s alpha (*α*) was also calculated for three factors of SfBS. An equal to or greater than 0.70 implies acceptable scale reliability (George and Mallery, 2003; Kline, 1999). Meanwhile, the composite scores of each subscale were determined by averaging the values of their components.

The EFA and CFA studies were computed using the lavaan package (Rosseel, 2012) for the R statistical computing environment (R Core Team, 2019), while the validity and internal reliability computation was performed using the SPSS 26.0 program.

## Results

### Exploratory factor analysis

Before the EFA was performed on Sample 1, each item’s skewness and kurtosis evaluation were examined. All items were within the range of ±1; this supported the assumption of normal distribution of this dataset (Bryman and Cramer, 2001; Kline, 2010). The item-total correlations were also satisfactory, following Wu (2010) suggested criteria (*r* > 0.40, *p* < 0.01). This indicated that Sample 1 was suitable for EFA testing. The Kaiser-Meyer-Olkin measure of sampling adequacy was 0.953, and Bartlett’s test of sphericity was χ^2^(136) = 10143.701, *p* < 0.001, implying that the data was acceptable for factor analysis.

Three factors with eigenvalues over 1.00 were identified and could be interpreted. The screen test (Cattell, 1966) echoed this solution further. The identified three-factorial structure of the self-feedback behavior model was aligned with the self-feedback behavioral model. However, three items did not produce an acceptable level of 0.40 on any factor; for example, item “*I reflect on the quality of my own work and use my reflection as a source of information to improve my work*” was deleted as it appeared that students do not think the self-reflection as a source of feedback, they would more prefer to seek external feedback from other people or their learning environment; item “*When other people provide me with input about my work I listen or read thoughtfully*” was deleted as students might interpret this item with emphasis on their listening and reading behavior. Therefore, they did not interpret this item as seeking feedback behavior; the item “I would spend more time working on my advantageous areas” was deleted since students would prefer spending more time on their weak areas for improvement rather than maintaining their advantageous areas. Meanwhile, another three items were reported to have cross-loading effects on both PF and UF. These three items were (*I consider how comments relate to criteria or standards; I consider my experience on similar tasks when doing my current work; I explicit the inferences after comparing comments with my learning objectives.*) Interestingly, this finding was consistent with some discussions raised in the focus group discussion where some students pointed out that they interpreted these three items as Use Feedback behavior. At the same time, some thought it should belong to the Process Feedback behavior. Eventually, the three-factorial solution based upon 11 items (Table 3) was obtained, namely, Seek Feedback (SF) with four items, Process Feedback (PF) with three items, and Use Feedback (UF) with four items. The three-factor solution contributed to 70.9% of the total variance.

**Table 3:** EFA factor loadings for each item (values below 0.3 are hidden).

	SF	SF	UF	Uniqueness
Item 2	0.801			0.212
Item 3	0.719			0.346
Item 5	0.700			0.261
Item 6	0.609			0.278
Item 9		0.840		0.183
Item 8		0.828		0.161
Item 7		0.683		0.186
Item 17			0.726	0.238
Item 16			0.700	0.271
Item 13			0.697	0.293
Item 15			0.418	0.499

Applied rotation method is oblimin. SF, seek feedback; PF, process feedback; UF, use feedback.

### Confirmatory factor analysis

Two CFAs were employed in Sample 2 based on the hypothesized model (model 2) to cross-validate the factorial structure (model 1) proposed by the EFAs. For model 1, CFA has produced a satisfactory model fit, as described in Table 4. For model 2, a series of CFAs were conducted, and an adequate model fit was achieved (Hu and Bentler, 1999; Wu, 2010). Five items were removed due to either their low factor loadings (less than 0.3) or high modification indices (more than four, resulting in a poor goodness-of-fit index). Eventually, model 2 consisted of 12 items and achieved an acceptable model fit. In conclusion, the cross-validation study for both models supported the three-factorial structure of SfBS, which aligns with the theoretical description of the self-feedback behavioral model.

**Table 4:** Invariance test across students of different genders.

	χ^2^	df	χ^2^/df	*P*	RMSEA	PNFI	CFI	Δ CFI	Δχ^2^	Δdf
M1	235.934	82	2.877	<0.001	0.078	0.706	0.965	–	–	–
M2	240.229	90	2.669	<0.001	0.074	0.774	0.966	0.001	4.295	8
M3	245.203	98	2.502	<0.001	0.070	0.842	0.966	0.000	4.974	8
M4	256.678	109	2.355	<0.001	0.066	0.934	0.966	0.000	11.475	11

Notably, due to our study’s comparatively large sample size (*N* = 626), the Chi-square indices were not ideal, as the χ^2^/df indices for both models were slightly more than 3.0. Therefore, we chose to evaluate the model fit by considering the rest of the model fit indices (Browne and Cudeck, 1993). Meanwhile, for model comparison purposes, the information criteria (e.g., Akaike’s Information Criteria, AIC, Bayesian Information Criteria, BIC) would meaningfully indicate model comparison for further selection. As Kline (2010) suggested, a smaller AIC and BIC implies a better model fit.

**Figure 2:**
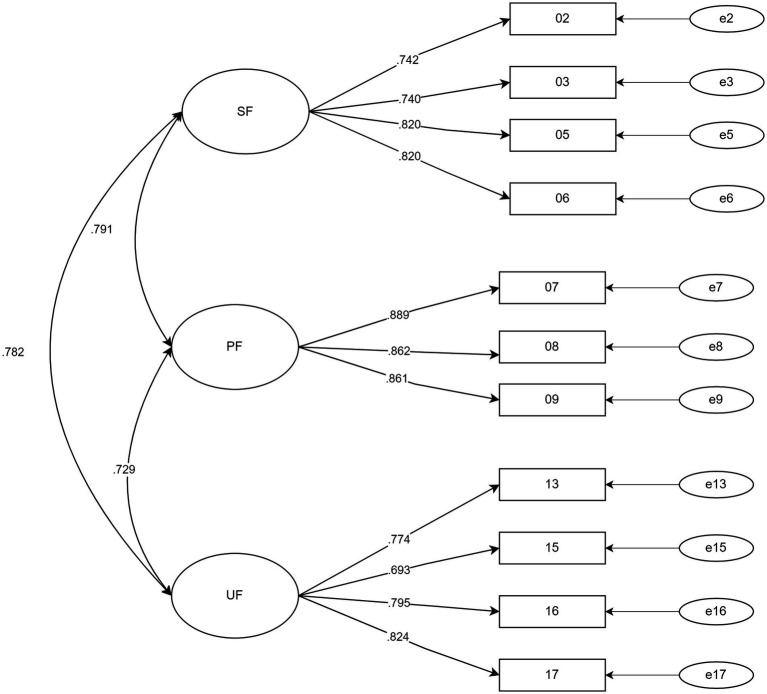
Confirmatory factor analysis item loadings and correlations for the final model (Model 1).

Table 2 reported the goodness-of-fit indices for both CFA models. Albeit both models obtained acceptable model fits, the CFI and TLI indices were greater than 0.90, RMSEA were less than 0.80, and SRMR was less than 0.50. However, model 1 achieved comparatively better regular model fit indices. Moreover, both AIC and BIC indices for model 1 were smaller compared with model 2. Therefore, Model 1 with 11 items was chosen as the final model, as described in Figure 2.

### Multi-groups CFAs

Multi-group CFAs were conducted for male and female participants. The chi-square test for variance should be stringent (Cheung and Rensvold, 2002). Considering the comparatively large sample size, the changes in CFI were adopted as criteria for evaluating model differences. A decrease of 0.01 in CFI could be acceptable to determine a lack of invariance across different groups (Byrne, 1998).

Table 4 reports the multi-group CFA results in different constraint levels. First, no equality constraints were imposed; the results indicated that M1 fit well with the CFI, RMSEA, and PNFI. Equality constraints were imposed on the measurement weights. M2 results also showed a good fit as the change of CFI was 0.001, implying the measurement weights in both gender groups are consistent. Equality constraints were further imposed on the measurement weights and measurement intercepts. No change of CFI was reported in M3. Finally, equality constraints were imposed on the measurement weights, intercepts, and structural covariances. Still, no change in CFI was reported in M4. In conclusion, these multi-group CFA tests suggested the consistency of the structure of SfBS across different gender groups.

### Validity

Table 5 reports the correlations between the three subscales of the SfBS and the five subscales of the CBF-PI-15 (Zhang et al., 2019). It was found that all three sub-scales of self-feedback behavior (SF, PF, and UF) had no significant correlation with neuroticism (N) but had substantial correlations with Conscientiousness, Agreeableness, Openness, and Extraversion (from 0.15 to 0.73, *p* < 0.05). It indicated that whether students had neuroticism personality or not, no impact was produced on their self-feedback behavior. Moreover, students with a low level of neuroticism were more likely to be active in seeking and processing feedback. In contrast, students’ conscientiousness was strongly correlated with self-feedback behaviors. Conscientiousness reflects thoroughness, responsibility, self-motivation, achievement orientation (Barrick and Mount, 1991; Costa and McCrae, 1992; Goldberg, 1993), and cooperation (Molleman et al., 2004). Individuals with high conscientiousness are more purposeful and motivated to accomplish tasks (Witt et al., 2002), suggesting that such students are more proactive in seeking, processing, and using feedback. Agreeableness, linked to compassion and cooperation, was also positively correlated with self-feedback, indicating that agreeable students are more likely to engage in the feedback process (Laursen et al., 2002). Openness, which measures creativity and receptiveness to new experiences, encourages students to seek and use feedback to enhance their performance (Schretlen et al., 2010). Extraversion, reflecting sociability and outgoingness, suggests that extroverted students are more likely to interact with teachers and peers to elicit feedback and apply it to their learning (Godfrey and Koutsouris, 2023).

**Table 5:** Means, standard deviations, and correlations for external validity.

CBF-PI-15						SfBS		
	N	C	A	O	E	SF	PF	UF
N	–							
C	0.005	–						
A	−0.021	0.223*	–					
O	0.039	0.266**	0.331**	–				
E	0.006	0.074	0.304**	0.271**	–			
SF	−0.038	0.615**	0.223**	0.359**	0.186*	–		
PF	−0.009	0.547**	0.160*	0.193*	0.149*	0.728*	–	
UF	0.069	0.567**	0.212**	0.237**	0.177*	0.572**	0.580**	–
M	2.942	4.019	3.981	4.056	3.517	4.161	4.265	3.970
SD	1.155	1.014	1.102	1.216	0.586	0.949	0.962	1.019

N, euroticism; C, conscientiousness; A, agreeableness; O, openness; E, extraversion; SF, seek feedback; PF, process feedback; UF, use feedback. *p < 0.05, **p < 0.01.

Furthermore, the convergent and discriminant validities of SfBS were also evaluated. All items demonstrated an acceptable range of factor loadings from 0.69 to 0.89, which implies robust convergent validity at the item level. The heterotrait-monotrait ratio of correlations (HTMT) analysis, which examines the ratio of the inter-item correlations between constructs to the inter-item correlations within a construct, was employed to evaluate the discriminant validity. Values of 0.85 or less are acceptable (Henseler et al., 2015). All three factors reported adequate discriminant validity in this study. In short, the factors were meaningfully different, and the strong correlations among the factors did not result from cross-loading items (Table 6).

**Table 6:** Means, standard deviations, HTMT ratio of correlation values among three factors for SfBS.

	SF	PF	UF
SF	–		
PF	0.776**	–	
UF	0.791**	0.735**	–
M	4.161	4.265	3.97
SD	0.949	0.962	1.019

SF, seek feedback, PF, process feedback, UF, use feedback. *p < 0.05, **p < 0.01.

### Reliability

The Cronbach’s alpha (*α*) reliability of the three factors was computed to vary between 0.85 and 0.90 (Table 7). All these values were above the criteria of acceptable reliability of 0.70. Therefore, the SfBS (see Appendix) was considered to have sufficient remaining items.

**Table 7:** Internal consistency reliability (Cronbach’s α).

	Internal consistency
SF	0.860
PF	0.904
UF	0.854
Total	0.925

SF, seek feedback; PF, process feedback; UF, use feedback.

## Discussion

This study aimed to develop and validate an instrument for assessing student self-feedback behavior using samples of mainland Chinese students. The results supported the Student Self-feedback Behavior Scale (SfBS) as an appropriate instrument to measure self-feedback behavior for high school students in mainland China. EFA and CFA supported a three-factor model representing seeking, processing, and using feedback actions. Multi-group CFA ensured measurement invariance across gender groups, indicating measurement consistency of the SfBS among male and female students.

This study has two limitations. First, all participants were drawn from a single cultural context, namely mainland China, where Confucian values are deeply embedded. These cultural norms likely shape students’ self-feedback behaviors, as they tend to show deference to teachers’ feedback (Hofstede, 2001). In contrast, students from Western cultures may be more inclined to question teachers’ feedback and rely on their own learning experiences (Joy and Kolb, 2009; Holtbrügge and Mohr, 2010). Consequently, future research should explore self-feedback behaviors across diverse sociocultural contexts to determine whether the items in the SfBS exhibit cross-cultural invariance. Second, while this study provides preliminary evidence for the content, substantive, structural, and generalizability aspects of the SfBS’s validity, it did not address the consequential dimension of validity. As Panadero et al. (2024) argue, students’ active participation in the self-feedback process can “have a stronger effect on performance and learning” (p. 4). Therefore, future studies should further investigate this dimension to enhance the assessment of self-feedback practices using the SfBS and examine its relationship with students’ academic performance.

Despite areas for improvement, the SfBS offers a valuable instrument for researchers investigating student self-feedback behavior. Its theoretical foundation aligns with the proposed self-feedback process and enables the collection of crucial data for constructing a comprehensive understanding of student actions in the self-feedback process. Enhanced insight into students’ self-feedback behavior can inform teaching practices, promoting self-feedback and optimizing its effects on learning outcomes.

From the teachers’ perspective, understanding how to effectively implement self-feedback as an instructional strategy in the classroom can illuminate its benefits and foster active student engagement in the feedback process. Additionally, from the students’ viewpoint, being equipped with self-feedback as a learning strategy can significantly reduce the likelihood of misinterpretations of feedback. This, in turn, enables students to make more nuanced evaluative judgments of feedback and enhances their feedback self-efficacy (Carless and Boud, 2018; Panadero et al., 2019b). Moreover, students can derive more significant benefits from the self-feedback process by taking informed actions, such as feed-back, correcting misconceptions or errors in assignments, adjusting their learning objectives through feed-up, and refining their learning improvement strategies through feed-forward (Carless and Boud, 2018; Hattie et al., 2021; Mandouit and Hattie, 2023).

## Conclusion and implications

This study made a theoretical contribution to the in-depth knowledge of the behavioral model of self-feedback. This self-feedback behavioral model can help researchers and teachers better understand how students could take the initiative to engage in the self-feedback process, thus offering insights into effective classroom instruction strategies. Furthermore, the SfBS provides a reliable instrument for researchers and practitioners to investigate students’ self-feedback behavior and its relevant areas of interest further. Researchers can use the SfBS to collect essential data measuring students’ self-feedback actions. With a clear description and understanding of students’ self-feedback, researchers and practitioners can better help students engage and benefit from their self-feedback process and, eventually, improve their academic self-efficacy and achievement.

## Data Availability

The raw data supporting the conclusions of this article will be made available by the authors, without undue reservation.

## Appendix

### Self-feedback Behavior Scale (SfBS)

#### Seek feedback (SF)

1. I seek out examples of good work to improve my work.

2. I ask for comments about specific aspects of my work from others.

3. When I am working on a task, I consider comments I have received on similar tasks.

4. I seek feedback information from various learning resources.

#### Process feedback (PF)

5. I carefully consider comments about my work before deciding whether to use them.

6. When receiving conflicting information from different sources, I judge what I will use.

7. When deciding what to do with comments, I consider the credibility of their sources.

#### Use feedback (UF)

8. I can formulate my learning improvement plan after explicit inferences.

9. I would spend more time working on my weak areas.

10. I plan how I will use feedback to improve my future work, not just the immediate task.

11. I would continue seeking comments to improve my future learning.
